# Every Day Practice

**Published:** 1886-07

**Authors:** E. G. Betty


### Every Day Practice.

BY E. G. BETTY, D.D.S.

(Read before the Mad River Dental Society.)

The second subject on the programme under the above caption means a great deal, and allows the widest latitude to any one desiring to present anything to this society. That being understood, I should like to offer a few suggestions, to start the discussion, which, I doubt not, will be profitable and interesting. I refer to the treatment of the average mouth as we see it for the first time, and make arrangements for the correction of such faults or diseases as may need our assistance to induce as perfect a condition as possible. It is not infrequently the case that the first glance into the mouth of young or old, male or female, that, we find a condition of things that is not at all appetizing either to the patient or the operator. The teeth here and there are covered with incrustations of salivary calculus, agglutinated mucous particles of food of various sorts in an advanced stage of decomposition, the soft parts in a chronic or semi-active state of inflammation, two or three odd roots abscessed in the socket, an aching pulp or two, and the dear only knows what else to augment the disagreeable list of odoriferous subjects.

Manifestly the very first thing to do is to thoroughly remove all extraneous matter of whatsoever kind, and prescribe some stimulating antiseptic wash, which will both improve the condition of the soft parts, and do away with the disagreeable odor upon the breath, that is so often the cause of the remark: "I cannot bear to talk to Mr. Smith, his breath smells so badly." Having well cleansed the mouth in such manner of detail as your intelligence will naturally suggest, you may then remove any useless or diseased roots there may be present, and institute preliminary treatment for the conservation of any exposed pulp or dead tooth, which, in your judgment, offers the least susceptibility of redemption. After all this has been done you may then turn your attention to those teeth requiring filling, and can go to work upon them, well assured that your patient readily appreciates your sense of cleanliness, and that your fine specimens of artistic reconstruction in gold will not be obscured in dirt at the risk of the loss of your reputation as an operator, and the refusal of the patient to requite the obligation in good hard dollars.

